# 70 years of wood modification with fungi

**DOI:** 10.1186/s40694-022-00136-9

**Published:** 2022-03-18

**Authors:** Stephanie Stange, André Wagenführ

**Affiliations:** Chair of Wood Technology and Fibre Materials Technology, Technische Universität Dresden, Dresden, Germany

**Keywords:** Fungal wood treatment, Myco-wood, Mycological wood modification, *Myko-Holz*, Walter Luthardt

## Abstract

To obtain special wood properties for various technical applications, fungi with their broad spectrum of activity can make a contribution. The foundations for today's mycological wood modifications were laid by researchers who wanted to increase the yield of edible mushrooms. They noticed the changed properties of the wooden substrate by the progressive wood degradation. Controlled use of fungi and an eye for the technical benefits of mycologically degraded wood revolutionized the fundaments of wood modification, primarily biological. In this context, the so-called *Myko-Holz* (myco-wood) plays a unique role and influences the current research for pencil wood, tone wood or even spalting.

## Background

Fungi are usually seen by the wood used only as destroyers or parasites. Material infestation should be avoided as far as possible for wood preservation reasons. Therefore, fungi serve the wood technology industry primarilyas test organisms in research and development work on the subject of wood protection to test fungicidal and fungistatic substances. However, the positive effects of wood colonizing fungi often remain unconsidered. Nevertheless, some scientists and companies that take advantage of the possibilities of wood biotechnology with fungi and their enzymes.

For instance, specific fungal cultures were used to modify or pretreat wood chips. This method is particularly suitable for the production of wood pulp and refining. During the defibering process, the fibers are split along the lignin-rich middle lamella; temperatures above the glass transition point of lignin prevail here, resulting in an inactive lignin crust forming on the fiber surface, which blocks the accumulation of binders. The mycological modification of wood chips partially degrades the lignin in wood. The remaining lignin is modified by fungi so that more binding phenolic hydroxyl groups are formed on the wood fibers. Thus, the use of additional binders in fiberboard production can be reduced. At the same time, energy savings of 30% to 50% could be achieved with this method in the defibering process with a low mass loss of 4% [1–4].

In studies on the pretreatment of wood chips with ligninolytic enzymes of fungal origin, it was shown that it is even possible to dispense entirely with synthetic binders in fiberboard production and adjust fiberboard properties by using fungal enzymes [4–6].

As given in the preceding examples, the idea of application of fungi in the wood industry can be traced back to the invention of the so-called *Myko-Holz* (Myco-wood) by Walther Luthardt. Through his work on technical-mycological wood loosening, basidiomycetes were explicitly used to improve wood properties. Thus, he permanently changed the bad reputation of fungi in the wood sector.

## The cultivation of edible mushrooms leads to the idea of *Myko-Holz*

The understanding that wood decay is related to fungal growth was first described by Hartig [7]. This realization was followed by a wide variety of research on fungus-specific wood decay that results in mass loss. Johannes Liese (1891–1952) established a test protocol to investigate the influence of fungal growth and resulting wood destruction, which sat the basis of the still valid wood preservation standards EN 113 and DIN 15083 [8]. In addition to the investigations on wood damage, J. Liese focused on the use of wood colonizing fungi for edible mushroom production [8] and thus laid the foundation for the development of *Myko-Holz*.

**Fig. 1:**
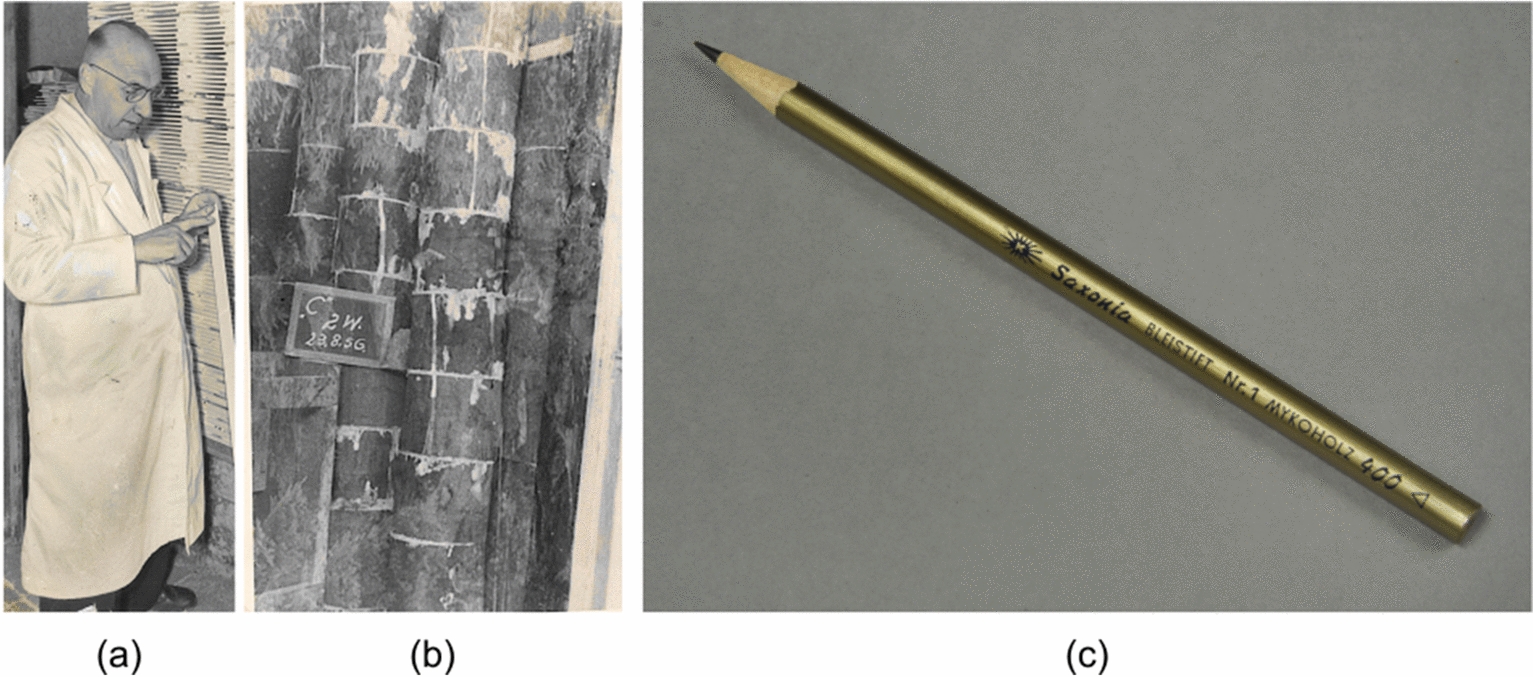
**a** Walter Luthardt with a scale bar made of Myko-Holz **b** production of Myko-Holz during the incubation process. **c** pencil made of Myko-Holz; photo. **a** and **b** provided by Helmut Luthardt

Furthermore, the engineer Walter Luthardt (Fig. 1a) investigated the living conditions of European wood colonizing fungi in Steinach (Thuringia, Germany). As early as 1949, while cultivating edible mushrooms such as the sheathed woodtuft (*Kuehneromyces mutabilis*), he noticed the coloration and properties of the logs on which the mushrooms were grown had changed. Due to the wood degradation by the fungus, the wood became lighter in color and weight and still had a relatively good cohesion in its internal structure [9–11]. Due to the resulting velvety wood surface, this wood found its first technical use as polishing wood in the watch industry [10]. Based on his observations, Luthardt developed an industrial process for technical-mycological loosening of wood intending to improve the mechanical workability of native hardwood, primarily European beech (*Fagus sylvatica* L.). Screening studies identified various wood-degrading fungal strains that grow as uniformly as possible in the longitudinal direction (fiber direction), with simultaneous low degradation of the transverse cell walls. In particular, the white-rot fungi oyster mushroom (*Pleurotus ostreatus*, edible) and polypore mushroom (*Trametes versicolor*, not relevant as an edible mushroom) were successfully tested in the laboratory and subsequently used for large-scale production of *Myko-Holz* [9].

This invention is based on the following definition: "*Myko-Holz* is wood which has been loosened by the controlled action of certain wood colonizing fungi and which has largely changed its technological properties or can acquire certain technical data as a result of controlled fungal decomposition" [10]. From this, the following guiding principle can be derived: "*Myko-Holz* is always a white-rotten wood—white-rotten wood is not always *Myko-Holz*!" [10].

The fungi as pure cultures were processed into a specially developed inoculation paste for its production, consisting mainly of wooden saw dust. This so-called "Tharandter inoculation paste" was applied to the cross-section of 0.4–1.2 m long, barked logs of beech as shown in Fig. 1b. The bark prevents the wood from drying out prematurely and, provides optimal growth conditions for the fungi in the log. The logs treated in this way were then stacked upright in dark rooms and stored in a warm, humid environment. The incubation period lasted three to six months [9, 10]. During incubation, the white rot fungi degraded the lignin and hemicelluloses in the wood preferentially and, the cellulose remained modified in the degraded wood behind [12].

To achieve sufficient strength or hardness at the edge zones (end faces), although the fungi reached the center of the wood logs, the degradation process was optimized by selecting fast-growing fungal strains and setting up optimal growth conditions e.g. temperature, moisture content or period of treatment [10, 13].

The fungal growth and the mycological degradation were stopped by cleaning the logs from the inoculation paste, followed by slow drying.

## Technology revolution through *Myko-Holz*

Shortage of raw materials in the German Democratic Republic (GDR) after the end of the second world war and the associated import stop of cedar wood from overseas paved the way for the *Myko-Holz* into various industrial sectors and especially into pencil production [10, 11]. Wood loosening changes the density (myco-beech: 0.13–0.45 g/cm^3^; native beech: 0.54–0.91 g/cm^3^) and the mechanical properties (E-modulus II for myco-beech: approx. 6700 N/mm^2^; native beech: approx. 16,000 N/mm^2^) of the wood [10, 14].

In general, wood mycologically modified by the Luthardt-method is characterized by the following attributes [10]:

- High and uniform porosity
- Low variation in raw density
- Stressless material (thus low warpage)
- Good insulating properties
- Low swelling
- Good heat and cold insulation
- High sound absorption
- High moisture absorption
- High absorption and good impregnability
- Good sharpenability

Due to this change in properties, especially due to the higher porosity and associated impregnability, the *Myko-Holz* can be used for a broad range of applications.

A wide variety of impregnating agents, such as waxes, paraffin, fire retardants and wood preservatives, are conceivable. While, for instance, fire retardants are used for highly flammable conveyor belts made of wooden slats, the *Myko-Holz* could be also hardened by impregnation with waxes for parquet flooring [9, 10].

As mentioned at the beginning, the use of wood for pencils is probably the most popular application of *Myko-Holz* (Fig. 1c).

As paraffin-impregnated *Myko-Holz* from beech has similar properties to the red cedar (*Juniperus virginiana*), which is used for pencil production, this imported wood can substitute by *Myko-Holz*. Besides, *Myko-Holz* has also been used to produce wooden molds for the glass industry, in model making or for foundry molds [10].

In the former GDR, annual production of approx. 120,000 L of inoculation paste and approx. 5000 solid cube meters of *Myko-Holz* (especially from beech) was achieved [9].

Walter Luthardt submitted applications for numerous patents related to the production and the use of *Myko-Holz* as given in Table 1. It can be concluded from his patents that the production of *Myko-Holz* originates from edible mushrooms production. In the process of mycological wood loosening, the core of Luthardt´s research was the proper preparation of inoculation.

**Table 1:** Overview of the Myko-Holz (myco-wood) related patents from Walter Luthardt

Year of application	Patent document	Content	Relation to *Myko-Holz*
1944	DD000000000292B1	[DE] Verfahren zur Züchtung holzbewohnender Pilze	Production of edable mushrooms, led to the invention of *Myko-Holz*
1951	DD000000003114A1	[DE] Verfahren zur Herstellung und Handhabung von Impfmaterial in der praktischen Pilzkultur	Preparation of the inoculation paste
1951	DD000000002175A1	[DE] Verfahren zur Veredelung von Holz durch Steigerung verschiedener Eigenschaften, wie die der Schnitz- und Spitzbarkeit, Feuerfestigkeit und Schwimmfähigkeit	Evaluation of potential applications of *Myko-Holz*
1953	DE000000946845B	[DE] Verfahren zur Veredlung von Holz	production of *Myko-Holz*
1953	DE000000932998B	[DE] Verfahren zur Herstellung und Handhabung von Impfmaterial in der Pilzkultur	Production of the inoculation paste
1957	DD000000022628A1	[DE] Schüttelkolben zur Anreicherung von Luftsauerstoff in Nährlösungen	Preparation of the inoculation paste
1958	DD000000021771A1	[DE] Glasform aus Holz	Application of *Myko-Holz* for glass industry
1959	DE000001063336A	DE] Schüttelkolben fuer biologische Zwecke, insbesondere zur Herstellung von Pilzkulturen	Preparation of the inoculation paste
1971	DD000000102553A1	[DE] Verfahren zur Herstellung von Pilzimpfmaterial zur Übertragung von Pilzkulturen, insbesondere für Kulturen holzbewohnender Pilze	Preparation of the inoculation paste

Some licenses for producing pencils from *Myko-Holz* were granted to the company Faber-Castell in 1953, which involved 110 countries (e. g. Brazil). In 1960, the "Luthardt process" was implemented at the KOH-I-NOOR pencil factory, CSSR [15]. With his patented processes and investigations, Walter Luthardt laid the foundation for the technical use of fungi in wood technology.

## *Myko-Holz*, nowadays

**Fig. 2:**
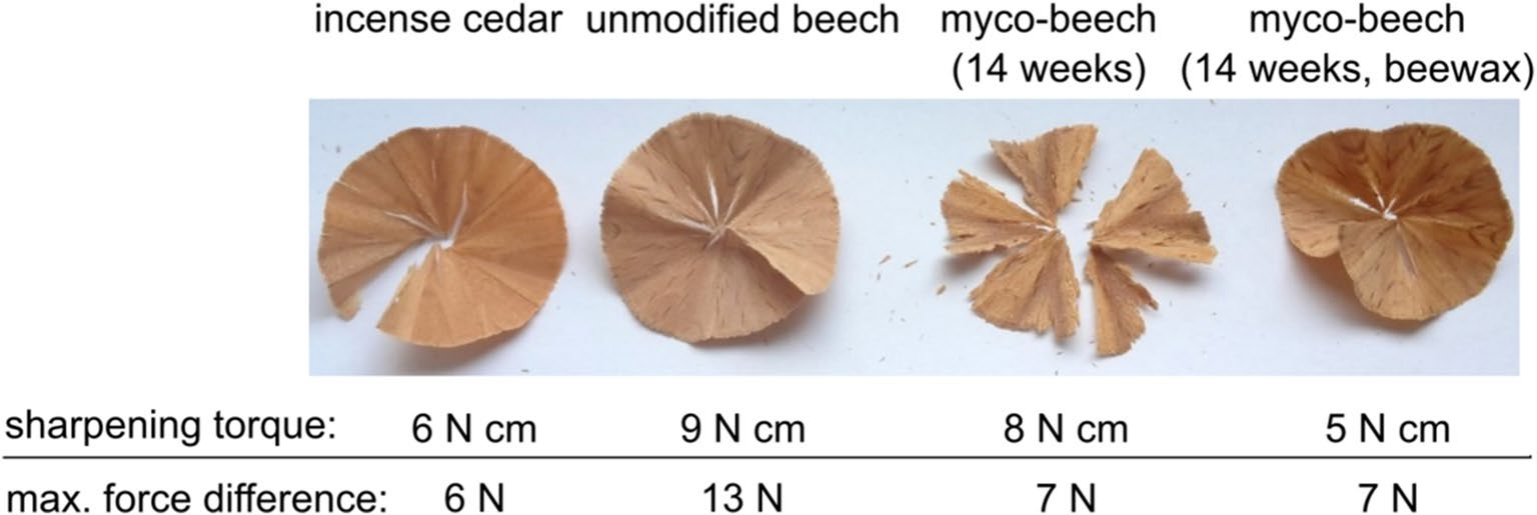
Comparison of sharpening chips and their characteristic values like sharpening torque and max. Force difference from the investigations of Oberer et al. [16]

To re-evaluate the properties of mycologically modified wood as an alternative to conventional pencil woods like incense cedar (*Calocedrus decurrens*) or Gmelina (*Gmelina arborea*) using modern methods, Oberer et al. [16] modified European beech (*Fagus sylvatica*) samples with *Trametes versicolor* according to Luthardt's experiments using different incubation periods. Subsequently, these samples were divided into two groups. Thereby, the control group remained untreated after mycological modification. Using vacuum pressure impregnation, a second group was impregnated with beeswax (solids content: 25%). Subsequently, the sharpening ability of the specimens was compared with the conventional pencil woods by determining the sharpening torque and the maximum force difference during the sharpening test. The determined characteristic values showed that similar values could be produced after 14 weeks of incubation for conventional pencil woods (Fig. 2). The sharpening result clarifies that due to the sole mycological modification, the resulting chip is very brittle. This quality deficiency was eliminated by impregnating the mycologically modified beech with beeswax. In summary, the investigations of Oberer et al. [16] showed that the unmodified beech is not suitable for wood-cased pins due to the high characteristic values (sharpening torque and max. force difference), but a mycological modification according to Walter Luthardt improves it [16]. These results confirm the value of Walter Luthardt's invention and its benefit for pencil production in the former GDR at the time.

**Fig. 3:**
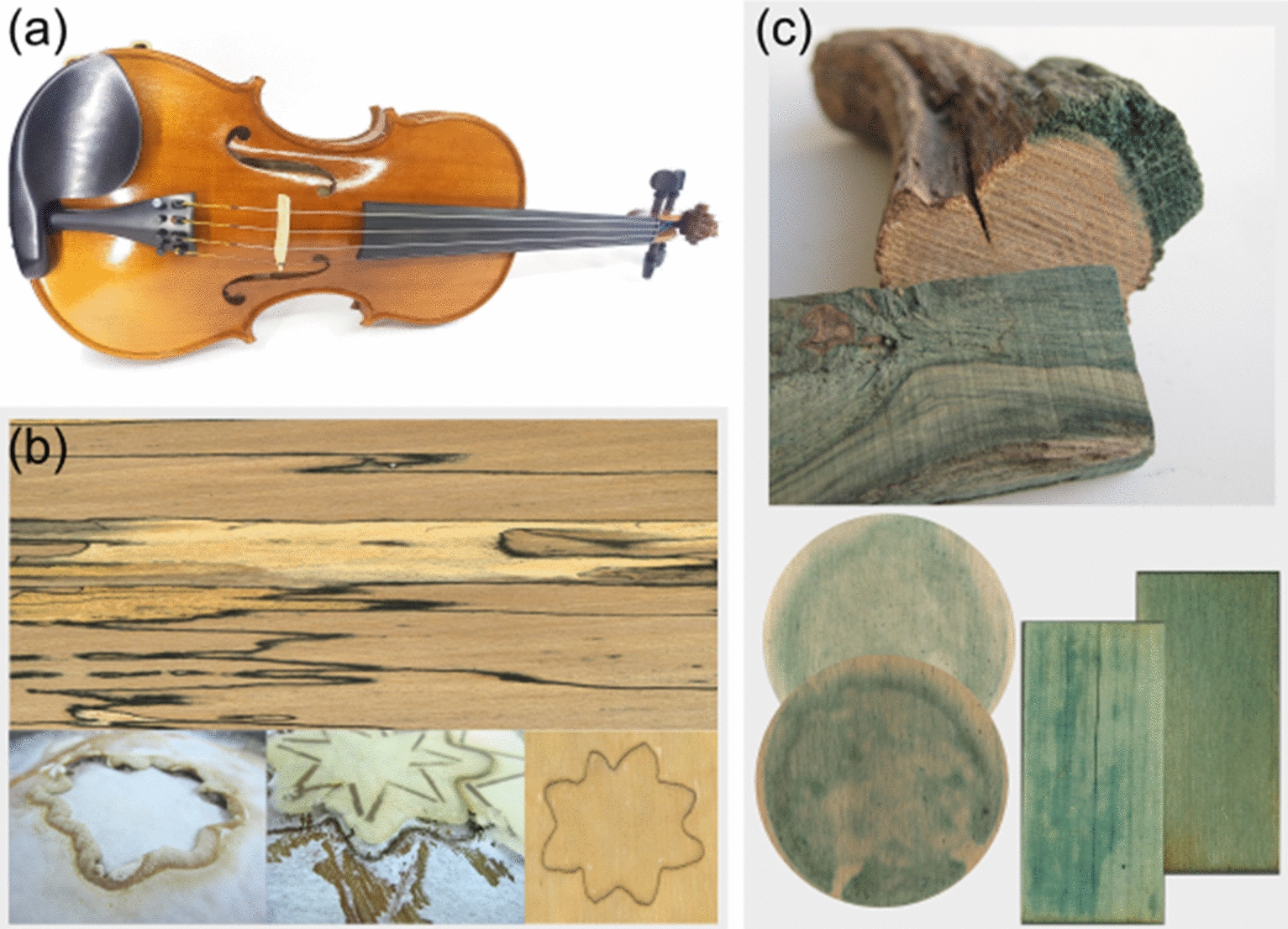
**a** Copy of a violin of Guarneri del Gesù **b** up: Truffle Beech veneer of Mehling and Wiesmann GmbH; down: production of controlled zone line pattern in dual cultures on birch veneer [24] **c** Pigmentation of Chlorociboria sp. in wood; up: naturally stained wood; down: controlled spalting in circular veneer [29, 31] and stained Myco-Holz [29, 30]

Not only the search for alternatives for pencil woods, but also the interest in wood, which sounds like the tonewood of the world-famous violins of Antonio Stradivari or Giuseppe Guarneri del Gesù (as given in Fig. 3a), found its way to Luthardt's invention. These master violin makers had disposal woods with unique wood properties and sound qualities. According to dendrochronological studies, the spruce of these world-famous violins comes from trees that grew during a small ice age ("Maunder Minimum") [17–19]. Thus, the wood has a uniform structure and narrow annual rings, which results in optimal sound velocity to raw density ratio in wood, leading to high tone quality for musical instruments. The scientists led by Francis Schwarze of EMPA (Switzerland) used mycological wood loosening to increase the tone quality of violin woods. For this purpose, they used *Xylaria longipes* to modify the violin back (made of maple – *Acer pseudoplatanus*) and *Physisporinus vitreus* for the wood of the violin top (made of spruce – *Picea abies*) [17, 18]. Both fungi belong to the white-rot fungi and decompose the wood uniformly, comparable to the production of *Myko-Holz,* according to Luthardt. With this mycological wood modification, the scientists around Schwarze achieved wood properties similar to those used for the violins of Stradivari and Guarneri del Gesù [18]. To prove the quality of the mycological tone wood, violins were made from it and tested. In a blind listening test, a mycologically modified violin won against an original Stradivarius violin [17].

*Myko-Holz* also forms the basis for mycological wood colorations such as spalting. Here, fungi are used to store their dyes and pigments in the wood.The fungal modification must be controlled as much as possible for this wood to be usable. Probably the best-known form of spalting is zone line formation. Here, dark three-dimensional melanin embeddings are created by the fungus in the wood, which become visible as a line in the cut or break pattern [20–22]. Research of the group of Sara C. Robinson [21, 23] and our research [20, 24] investigates the controlled embedding process of the dye in the wood. *Lentinus tigrinus* [20], *Trametes hirsuta* [20], *Trametes versicolor* [21] or *Xylaria polymorpha* [25] are particularly suitable for this type of modification. Growth and formation of the zone lines strongly depend on the species of wood and fungus used. Through growth control, patterns or lettering can be brought into the wood by zone line forming fungi (Fig. 3b) [24, 26]. This type of mycological modification is already used by Mehling und Wiesmann GmbH (Lohr am Main / Germany) to produce of decorative veneer. Their product, Truffle Beech (Trüffelbuche®), is sold worldwide as a luxurious premium veneer as shown in Fig. 3b [27, 28]. Particularly in arts and crafts, wood with zone lines finds great interest for e. g. wooden jewelry, vases, or bowls.

The color incorporation of the fungal pigment xylindein of *Chlorociboria* sp. is favored in white-rotten wood [25, 29–31]. As presented in our research and Fig. 3c [29, 30], beech (*Fagus sylvatica*) and birch (*Betula pendula*) were modified with different white rotten fungi according to the preparation of *Myko-Holz*. Especially beech pretreated with e. g. *Pleurotus eryngii* or birch pretreated with *Bjerkandera adusta*, *Lentinula edodes*, *Lentinus tigrinus* and *Trametes versicolor* showed good staining results due to a secondary colonization with *Chlorociboria aeruginascens* [29, 30]. These modification methods may eventually create ecologically sustainable alternatives for tropical woods in the future.

## Conclusions

The invention of *Myko-Holz* (myco-wood) by Walter Luthardt and thus, the technical application of fungus-treated wood fundamentally changed the attitude of wood technologists to fungi. Since then, they are no longer considered harmful organisms but also as a medium for modification to improve wood properties. To reach the technical usage of the wood, Luthardt used white rot fungi under controlled treatment conditions. The wood modified in this way is characterized, for example, by a more porous structure, a lower density and improved impregnability, which makes wood versatile in its specific application. Due to its similar properties and the shortage of raw materials especially in the former GDR, red cedar was substituted by paraffin-impregnated *Myko-Holz* from beech as pencil wood.

*Myko-Holz* and its numerous patented process and manufacturing methods, mainly focusing on the technical benefits of mycologically degraded wood, revolutionized biological wood modification and still influences wood technology research and industry today. Researchers improved, for instance, the sound qualities of spruce and maple by a controlled mycological modification [18]. Even the controlled spalting like zone line production or pigmentation, which is in the interest of research and industry, follows the methods and goals of Walter Luthardt.
